# Development and evaluation of an assay for the detection of tick-borne encephalitis virus RNA via real-time PCR with reverse transcription

**DOI:** 10.1186/s13071-026-07366-5

**Published:** 2026-03-23

**Authors:** Alena Sharova, Marina Safonova, Anna Dolgova, Anna Shabalina, Margarita Popova, Tatiana Arbuzova, Svetlana Schirobokova, Anna Gladkikh, Dmitry Naydenov, Valeriya Sbarzaglia, Ekaterina Klyuchnikova, Ivan Kholodilov, Galina Karganova, Edward Ramsay, Vladimir Dedkov

**Affiliations:** 1 Saint Petersburg Pasteur Institute, Saint Petersburg, Russia; 2 Anti-Plague Center, Federal Service on Consumers’ Rights Protection and Human Well-Being Surveillance, Moscow, Russia; 3 Chumakov Federal Scientific Center for Research and Development of Immune and Biological Products of the RAS (Institute of Poliomyelitis), Moscow, Russia; 4 Institute of Translational Medicine and Biotechnology, Sechenov First Moscow State Medical University, Moscow, Russia; 5 Martsinovsky Institute of Medical Parasitology, Tropical and Vector Borne Diseases, Sechenov First Moscow State Medical University, Moscow, Russia

**Keywords:** Tick-borne encephalitis virus, TBEV, *Orthoflavivirus encephalitidis*, RT‒PCR, Russia

## Abstract

**Background:**

Tick-borne encephalitis virus (TBEV, *Orthoflavivirus encephalitidis*) is an arbovirus of the family Flaviviridae. It is the etiological agent of tick-borne encephalitis (TBE), a severe disease affecting the central nervous system. Among arboviral infections, TBE represents the greatest burden in northern Eurasia, both in terms of emerging infection risk and mortality. Globalization and climate change increase the risk of TBEV introduction into nonendemic countries. They may also lead to the emergence of new viral variants featuring increased virulence for humans or altered antigenic characteristics. Hence, sensitive and specific TBEV detection methods are needed not only for diagnostics but also for One Health approach goals (surveillance and identification of viral sources in the environment).

**Methods:**

Here, we describe a newly developed reverse transcription PCR (RT‒PCR) assay for TBEV detection. The assay was developed and evaluated using armored RNA positive control particles (ARCs). The assay was evaluated using several sample types: (1) a panel of heterologous viral and bacterial RNA/DNA; (2) RNA from TBEV strains isolated in different years in various Russian regions; and (3) RNA from TBEV-positive and TBEV-negative ticks (collected in northwest Russia).

**Results:**

The limit of detection (LOD) of the assay is 10^3^ copies/mL (20 copies/reaction) of TBEV RNA. The developed demonstrated 100% analytical specificity. The assay was compared with the two most commonly used Russian commercial kits for TBEV diagnostics.

**Conclusions:**

The results indicate that the developed RT‒PCR assay is a reliable and competitive method for the detection of TBEV RNA, which establishes its value as a tool for diagnosing and monitoring the virus.

**Graphical Abstract:**

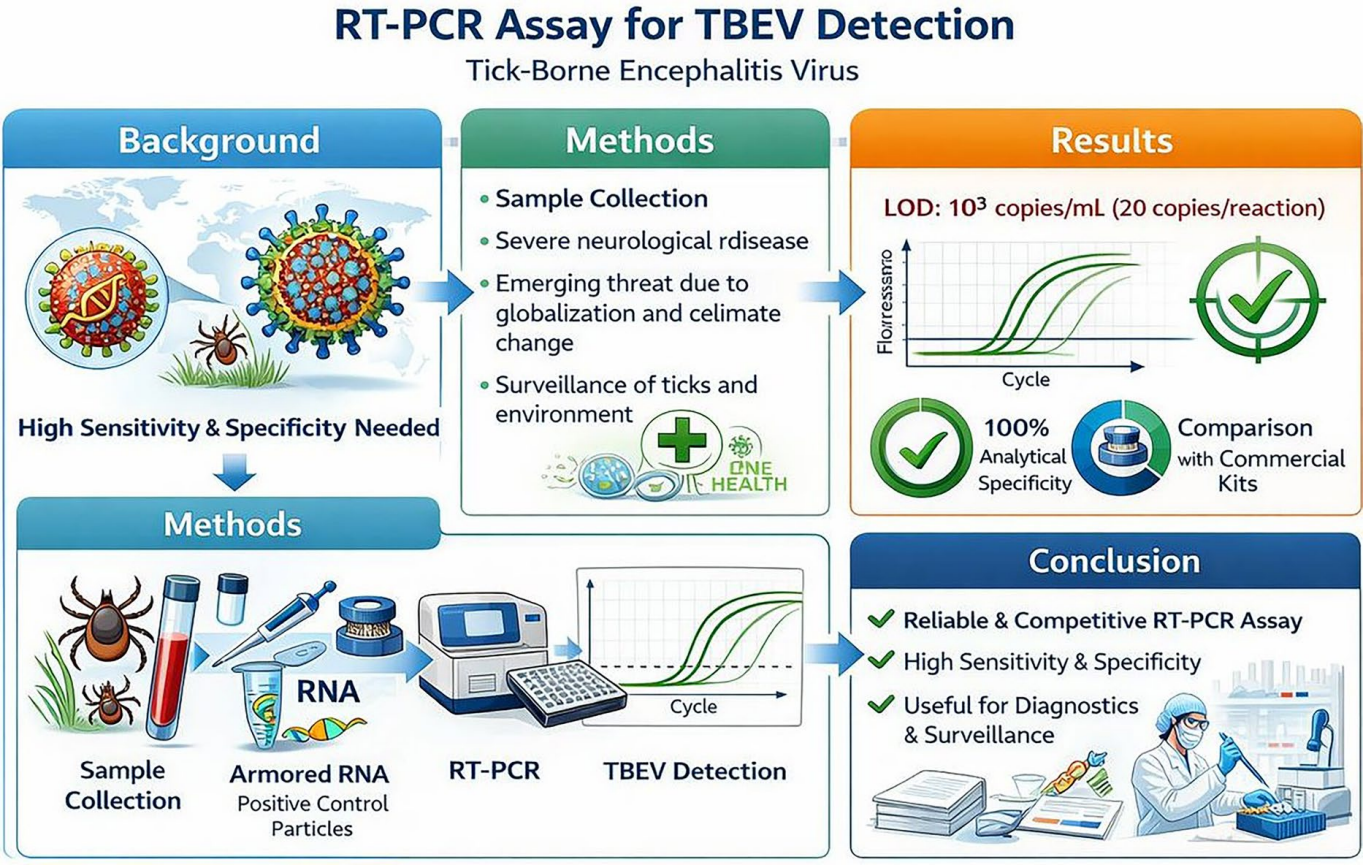

**Supplementary Information:**

The online version contains supplementary material available at 10.1186/s13071-026-07366-5.

## Background

Tick-borne encephalitis virus (TBEV, *Orthoflavivirus encephalitidis*) is a neurotropic arbovirus of the family Flaviviridae. It is the etiological agent of tick-borne encephalitis (TBE), a severe disease affecting the central nervous system [1]. The primary vectors for TBEV are ticks of the *Ixodes* genus: *Ixodes ricinus* in Europe and *Ixodes persulcatus* in Asia. [2–4]. TBEV’s range spreads widely from the Bordeaux region of France in the west through Italy in the south and the Scandinavian Peninsula in the north to Siberia, China, and Japan in the east [5]. The virus has been increasingly reported in new endemic regions, with its geographic spread influenced by climate change, ecological shifts, and human activities [6].

TBEV remains the most prevalent arbovirus in Europe. The reported incidence ranged from 0.4 to 0.9 cases per 100,000 people between 2015 and 2020 [7]. In Russia, the reported TBEV incidence was 1.3 cases per 100,000 people in 2024. This is slightly lower than the average incidence (1.7 cases per 100 K) for the 2015–2024 observation period [8]. There are at least 49 Russian regions where TBEV is prevalent. Among these regions, TBE incidence is classified as follows: 16 are recognized as high risk (5.1 per 100 K, average); 15 are recognized as moderate risk (1.6 per 100 K, average); and 18 are recognized as low risk (0.23 per 100 K, average).

TBEV virions measure approximately 50 nm in diameter and feature a nucleocapsid surrounded by a host-derived lipid membrane embedded with viral envelope (E) and membrane (M) proteins. The viral genome consists of a single-stranded, positive-sense RNA molecule (~10.2 kb) containing a single open reading frame (ORF) that encodes a polyprotein precursor [9]. This precursor is cleaved into three structural proteins (C, prM, and E) and seven nonstructural proteins (NS1, NS2A, NS2B, NS3, NS4A, NS4B, and NS5) [10].

According to the current consensus, TBEV is classified into seven main genotypes: European (TBEV-Eu), Siberian (TBEV-Sib), Far Eastern (TBEV-FE), Baikalian (TBEV-Bkl-1, TBEV-Bkl-2) Himalayan (TBEV-Him), and Ob (TBEV-Ob) [11]. TBEV-FE is prevalent in the Russian Far East and northern China. TBEV-Eu is dominant in Western, Central, and Eastern Europe [12]. TBEV-Bkl-1 and TBEV-Bkl-2 have been found in eastern Siberia near Lake Baikal and northern Mongolia [13, 14]. TBEV-Ob was found in western Siberia in the vicinity of the Ob River [15]. The TBEV-Him genotype was found in the Tibetan Highlands of China [16]. TBEV-Sib is the most common genotype and has been found in all regions where TBEV has been detected [12]. TBEV-Sib is represented by three phylogenetic lineages: *Baltic* (TBEV-Sib^*Balt*^), *Asian* (TBEV-Sib^*Asia*^), and *South-Siberian* (TBEV-Sib^*S.−Sib*^) [14]. Each genotype exhibits distinct pathogenic characteristics influenced by genetic variation. These factors affect virulence, immune evasion, and transmission potential [12]. The virulence of different TBEV genotypes varies significantly. The ranking by case fatality rate among the three traditional genotypes (highest to lowest) is as follows: Far Eastern (5.0–20.0%), Siberian (6.0–8.0%), and European (0.5–2.0%) [17]. Currently, there is no conclusive evidence that the TBEV-Ob, TBEV-Bkl-1/2, or TBEV-Him genotypes infect or cause disease in humans [15, 18].

The geographical distribution of TBEV has undergone significant changes owing to rising temperatures, changes in tick habitats, increased tick‒host interactions, and changes in bird migration patterns that favor viral spread to new geographical locations [19]. Since the turn of this century, novel TBEV endemic areas have been identified in Bosnia [12], the Netherlands, England [20, 21], South Korea [22], Mongolia, Denmark, high-altitude Kazakhstan, Kyrgyzstan, parts of Armenia, Azerbaijan, Uzbekistan [3], Austria [23], Moldova [24], and the Moscow region (Russia) [25].

The spread of TBEV to new regions is associated with the expansion of the areas of its main vectors, *I. ricinus* and *I. persulcatus*. This process may be associated not only with a change in the boundaries of TBEV transmission but also with a change in viral properties. The range of vertebrates involved in viral circulation may differ in the new region, and related changes in the vector are also possible [26].

A good example is the Baltic group of the Siberian TBEV subtype. Currently, regions in northwest Russia, the Baltic, and Scandinavian countries are actively populated by *I. persulcatus*, which carries the Siberian viral subtype [27]. The northwestern region of Russia includes regions with different risk levels: high (Vologda and Arkhangelsk regions, Republic of Karelia), moderate (Republic of Komi, Kaliningrad, Leningrad and Pskov regions, St. Petersburg), and low (Novgorod region) [8]. TBEV-Sib^*Balt*^ is dominant [28]. TBEV-Sib^*Balt*^ has also been found in central Russia (Yaroslavl region), the Ural region (Sverdlovsk, Kurgan regions) [29–31], and certain Baltic countries (Finland, Estonia, Latvia) [32–34].

Given its medical significance, tick-borne encephalitis necessitates an ongoing epidemiological surveillance program similar to those implemented in Russia and European countries. According to the European Centre for Disease Prevention and Control (ECDC) and national epidemiological surveillance systems, a comprehensive TBE monitoring program is in place within the European Union and the European Economic Area (EU/EEA). This program was institutionalized at the supranational level following the inclusion of TBE in the list of diseases subject to mandatory reporting in the EU surveillance system in 2012 [35]. An important component of the implementation of these programs is the availability of reliable molecular methods, such as reverse transcription–polymerase chain reaction (RT‒PCR), which can be employed to detect TBEV RNA [7]. In this context, it is important to develop new assays and evaluate existing ones for performance. Evaluations preferably include a large set of TBEV strains and field samples to assess the ability to detect all circulating viral variants.

Here, we report a newly developed RT‒PCR assay (TBEV AmpPS) for the detection of TBEV RNA, with a special emphasis on TBEV-Sib^*Balt*^, which is prevalent in northwest Russia. The developed assay was also compared with the two most commonly used Russian commercial kits for TBEV diagnostics.

## Methods

### Identification of conserved target sites for primer and probe design

Available complete genome sequences of tick-borne encephalitis virus (as of December 2024) were retrieved from GenBank (NCBI Nucleotide; *n* = 222) to identify conserved regions. A query targeting both the organism name and a sequence length within ±10% of reference sequences was used. To minimize sequence redundancy and ensure a representative sample, the dataset was curated for samples with ≤ 96% nucleotide identity using CD-HIT (v4.8.1) [36, 37], resulting in a curated set of 45 tick-borne encephalitis viruses. Their accession numbers are as follows: PV626569.1, PV173737.1, MG243699.1, PQ015165.1, PP473573.1, PP473570.1, OR896869.1, ON408073.1, ON408072.1, MW256716.1, MK560446.1, MT974474.1, MT228628.1, MN114637.1, MN115820.1, MN115819.1, MN115818.1, MH645613.1, NC_001672.1, LC440459.1, MH094241.1, MG599477.1, MG589940.1, MF774565.1, KT069219.1, KU761575.1, KU761569.1, LC171402.1, KX268728.1, KT001073.1, KP716978.1, KP716973.1, KJ922515.1, LC017693.1, KM019545.1, KJ739731.1, KJ701416.1, KJ626343.1, KF951037.1, JX534167.1, AB753012.1, FJ968751.1, EU816450.1, FJ402885.1, and EU816454.1.

**Fig. 1:**
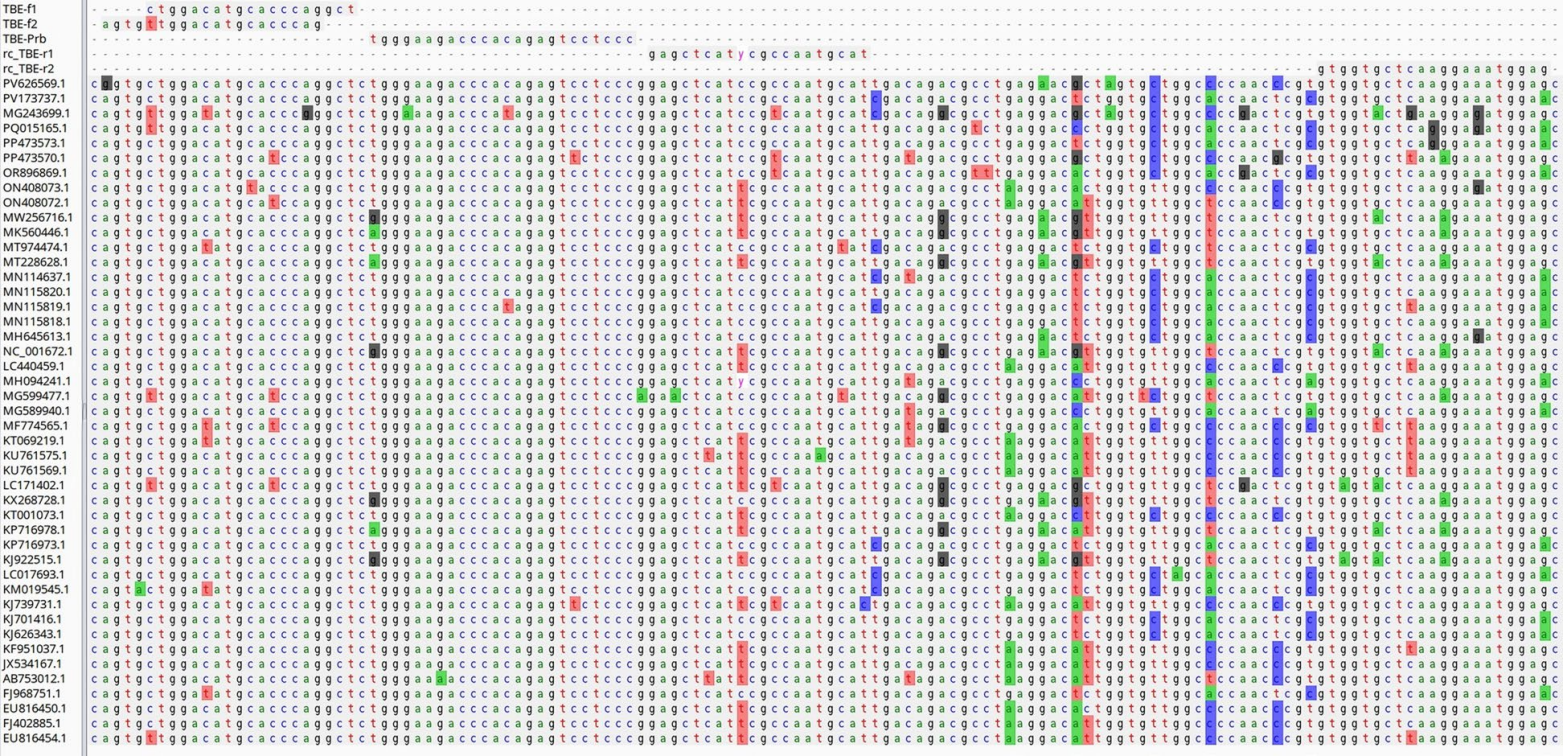
Partial nucleotide sequence alignment of the TBEV *NS3* gene

A local BLAST database was generated from this representative subset. The primer and probe sequences were aligned to the genome set via the BLASTN-short algorithm to determine binding sites. Multiple sequence alignment of the representative genomes, along with primer and probe oligonucleotides, was carried out via MAFFT (v7.450) under default settings [38]. An *NS3 gene* region fragment, 130 bp in length, was selected as the target for amplification via PLOTCON (http://emboss.bioinformatics.nl/cgi-bin/emboss/plotcon). The fragment represents nucleotide positions 5184–5113 in the TBEV reference sequence (GenBank accession number NC001672). The primers and probes were designed in accordance with the prevailing guidelines for RT‒PCR techniques (Fig. 1) [39].

The primer melting temperatures were calculated via the OligoCalc tool [40]. In addition, an oligonucleotide property calculator and MFOLD were used to assess the thermodynamic characteristics of the probe and the probability of secondary structure formation [41]. The primers and probes were synthesized by Genterra, PLC (Moscow, Russia). The primer and probe sequences are presented in Table 1.

**Table 1:** List of primers and probes used

Primer/probe	Sequence 5′–3′	Product size, bp	Probe type	Gene target	Length, nt	Position in ref seq (NC_001672)
TBE-f1	CTGGACATGCACCCAGGCT	63–130	TaqMan	NS3	19	5188–5206
TBE-f2	AGTGTTGGACATGCACCCAG				20	5184–5203
TBE-r1	ATGCATTGGCGRATGAGCTC				20	5233–5252
TBE-r2	CTCCATTTCCTTGAGCACCAC				21	5293–5313
TBE-Prb	R6G-TGGGAAGACCCACAGAGTCCTCCC-BHQ1				24	5208–5231
ICS_for	CCGGATTGCGTATCTCCGGACT	122	TaqMan	Artificial target	22	5188–5206
ICS_rev	CACGGCGGCATCTCTATCACGA				22	5184–5203
ICS_prb	FAM-TAGCTGGGCGTCAGGAATCCCAGG-BHQ1				24	5233–5252

### Positive and internal control preparation

The control sample set is similar to that already described [42–44]. It includes an internal extraction control (IC), an RNA control (ARC), a negative extraction control (NEC), and PCR controls (C^+^, C^−^). Briefly, the internal extraction control and armored RNA control are modified MS2 phage particles that include either an artificial sequence (internal extraction control, IC) or a sequence of the virus to be detected (armored RNA control, ARC). The positive PCR control (C^+^) contained a mixture of plasmids containing the same regions. The negative PCR control (C^−^) and negative extraction control (NEC) contained ultrapure water (Milli-Q, Merck KGaA, Darmstadt, Germany). A 130-nucleotide TBEV target fragment, which has 100% identity with primer and probe sequences (strain Nikolaevsk 855, TBEV-FE, GenBank ID: KP869172.1), was synthesized de novo via a previously described two-step PCR method [45]. Phusion high-fidelity DNA polymerase (NEB, Ipswich, MA, USA) was used with the primers listed (Table 2) to create cDNA and RNA controls.

**Table 2:** Oligonucleotides used for de novo cDNA synthesis by two-step PCR

Primer	Nucleotide sequence, 5′–3′	Position in sequence (KP869172.1)
TBE 1	AGTGCTGGACATGCACCC	5160–5177
TBE 2	GCGAATGAGCTCCGGGAGGACTCTGTGGGTCTTCCCAGAGCCTGGGTGCATGTCCAGCAC	5172–5231
TBE 3	CCCGGAGCTCATTCGCCAATGCATTGACAGACGCCTAAGGACGTTGGTGTTGGCCCCAAC	5205–5264
TBE 4	CTCCATTTCCTTAAGCACCACACGGGTTGGGGCCAACACCAA	5248–5289

### Reaction mixture composition and amplification conditions

The reaction mixture included the following components: 1 µL of BioMaster Mix (Biolabmix, Novosibirsk, Russia); 12.5 µL of 2× reaction buffer (Biolabmix, Novosibirsk, Russia); 5 pmol/reaction of each TBEV-specific primer (TBE-f1, TBE-f2, TBE-r1, TBE-r2); 5 pmol/reaction of the TBEV probe; 7 pmol/reaction of the internal control primers; and 5 pmol/reaction of the internal control probe (IC_f, IC_r, IC_prb). The total volume of primers and probes was 1.5 µL. The sample volume used was 10 μL, and the total reaction volume was 25 µL.

The optimized amplification program was as follows: 50 °C for 15 min and 95 °C for 5 min, followed by 40 cycles (95 °C for 10 s and 57 °C for 20 s). Fluorescence was recorded during the 57 °C step in the HEX/yellow channel (for TBEV) and the FAM/green channel (for IC). Reactions were performed via CFX96 C1000 Touch (Bio-Rad, Hercules, CA, USA) and Rotor-Gene Q (Qiagen, Hilden, Germany) PCR machines. The fluorescence threshold was set as the midpoint within the linear increase in positive-control fluorescence (log scale). Amplification was considered positive if the level of fluorescence crossed the threshold.

### Limit of detection

The limit of detection (LOD) was determined via a series of tenfold dilutions of armored RNA particles (ARCs, same as the positive control for reverse transcription) at known concentrations. The concentrations were measured via droplet digital PCR (ddPCR). The concentrations used to determine the limit of detection were 10^4^, 10^3^, 10^2^, and 10 copies of increased RNA particles per milliliter (200, 20, 2, and 0.2 copies/reaction, respectively). Samples from each dilution (100 µL) were extracted in triplicate via the RIBO-prep kit (AmpliSens, Moscow, Russia) according to the manufacturer’s instructions (elution volume 50 µL), followed by testing via the developed TBEV RT‒PCR assay (TBEV AmpPS). The LOD was set as the maximum dilution yielding a positive result in all replicates.

### Analytical specificity

The absence of nonspecific reactions with nucleic acids from other organisms was determined via a panel of heterologous viral and bacterial RNA/DNA. These solutions (*n* = 29) were obtained from the collections of the Saint Petersburg Pasteur Institute and the Institute of Poliomyelitis. The inclusion criteria were as follows: a virus or bacterium transmissible by ticks; viral members of the genus *Orthoflavivirus*; or viruses capable of causing CNS symptoms (TBE-like, flu-like) [7]. The microorganisms represented are presented in Table 3.

**Table 3:** List of viral species used in this study to evaluate nonspecific reactions

*N*	Species	Acronym	Family	Genus	Nucleic acid	Specific RT–PCR/PCR kit	*C*_t_ value	TBEV AmpS assay
1	*Ehrlichia* spp.	*Eh. *spp.	Ehrlichiaceae	*Ehrlichia*	DNA	AmpliSens TBEV, *B. burgdorferi* s.l., *A. phagocytophillum*, *E. chaffeensis*/*E. muris*-FL	30.1	Negative
2	*Rickettsia sibirica*	*R. sibirica*	Rickettsiaceae	*Rickettsia*	DNA	RealBest DNA *Rickettsia sibirica*/*Rickettsia heilongjiangensis*	21.5	Negative
3	*Coxiella burnetii*	*C. burnetii*	Coxiellaceae	*Coxiella*	DNA	AmpliSens *Coxiella burnetii*-FL	20.1	Negative
4	*Borrelia burgdorferi* sensu lato	*B. burgdorferi* s.l	Borreliaceae	*Borrelia*	DNA	AmpliSens TBEV, *B. burgdorferi* s.l., *A. phagocytophillum*, *E. chaffeensis*/*E. muris*-FL	32.1	Negative
5	*Anaplasma phagocytophilum*	*A. phagocytophilum*	Anaplasmataceae	*Anaplasma*	DNA	AmpliSens TBEV, *B. burgdorferi* s.l., *A. phagocytophillum*, *E. chaffeensis*/*E. muris*-FL	34.2	Negative
6	*Orthonairovirus haemorrhagiae*	CCHFV	Peribunyaviridae	*Orthonairovirus*	RNA	AmpliSens CCHFV-FL	15.4	Negative
7	*Orbivirus magninsulae*, genotype Kemerovo,strain 21/10	KEMV-21/10	Sedoreoviridae	*Orbivirus*	RNA	In house RT–PCR [46]	18.4	Negative
8	*Orbivirus magninsulae*, Tribec genotype, strain Tr-19	TRBV-Tr-19	Sedoreoviridae	*Orbivirus*	RNA	NA	–	Negative
9	*Orbivirus magninsulae*, Tribec genotype, strain Tr-25	TRBV-Tr-19	Sedoreoviridae	*Orbivirus*	RNA	NA	–	Negative
10	*Thogotovirus dhoriense*	DHOV	Orthomyxoviridae	*Thogotovirus*	RNA	NA	–	Negative
11	*Orthoflavivirus zikaense*	ZIKV	Flaviviridae	*Orthoflavivirus*	RNA	AmpliSens Zika virus-FL	32.3	Negative
12	*Orthoflavivirus flavi*	YFV	Flaviviridae	*Orthoflavivirus*	RNA	AmpliSens Yellow fever virus-FL	28.4	Negative
13	*Orthoflavivirus nilense*	WNV	Flaviviridae	*Orthoflavivirus*	RNA	AmpliSens WNV-FL	27.2	Negative
14	*Orthoflavivirus denguei* genotype 1	DENV 1	Flaviviridae	*Orthoflavivirus*	RNA	AmpliSens Dengue virus type-FL	24.7	Negative
15	*Orthoflavivirus denguei* genotype 3	DENV 3	Flaviviridae	*Orthoflavivirus*	RNA	AmpliSens Dengue virus type-FL	23.8	Negative
16	Influenza A/H1N3	FLUAV(H1N3)	Orthomyxoviridae	*Alphainfluenzavirus*	RNA	AmpliSens Influenza virus A-type-FRT PCR	18.4	Negative
17	Influenza A/H3N2	FLUAV(H3N2)	Orthomyxoviridae	*Alphainfluenzavirus*	RNA	AmpliSens Influenza virus A-type-FRT PCR	17.9	Negative
18	Influenza B	FLUBV	Orthomyxoviridae	*Betainfluenzavirus*	RNA	AmpliSens Influenza virus A/B-FRT PCR	23.6	Negative
19	Human coronavirus 229E	CoV 229E	Coronaviridae	*Alphacoronavirus*	RNA	AmpliSens ARVI—screen-FRT	24.9	Negative
20	Human coronavirus NL63	CoV NL63	Coronaviridae	*Alphacoronavirus*	RNA	AmpliSens ARVI—screen-FRT	31.4	Negative
21	Human coronavirus OC43	CoV OC43	Coronaviridae	*Betacoronavirus*	RNA	AmpliSens ARVI—screen-FRT	23.3	Negative
22	Severe acute respiratory syndrome coronavirus 2	SARS-CoV-2	Coronaviridae	*Betacoronavirus*	RNA	COVID-19Amp (St. Petersburg Pasteur Institute) [42]	17.3	Negative
23	*Orthopneumovirus bovis*	RSV B1	Pneumoviridae	*Orthopneumovirus*	RNA	AmpliSens ARVI—screen-FRT	21.9	Negative
24	*Mastadenovirus blackbeardi*	HAdV 3	Adenoviridae	*Mastadenovirus*	DNA	AmpliSens All screen-FRT	19.8	Negative
25	Human rhinovirus B (Type 17)	HRV-B	Picornaviridae	*Enterovirus*	RNA	AmpliSens ARVI-screen-FRT	24.4	Negative
26	Lyssavirus irkut	IRKV	Rhabdoviridae	*Lyssavirus*	RNA	NA	–	Negative
27	Lyssavirus rabies	RABV	Rhabdoviridae	*Lyssavirus*	RNA	RABV AmpPS [47]	18.3	Negative
28	Human alphaherpesvirus 1	HSV-1	Herpesviridae	*Alphaherpesvirus*	DNA	AmpliSens HSV I, II-FRT	21.8	Negative
29	Human Cytomegalovirus 5	HCMV-5	Herpesviridae	*Cytomegalovirus*	DNA	AmpliSens CMV-FRT PCR	19.9	Negative

To assess the ability of the developed assay to detect different TBEV genetic variants prevalent in Russia, an RNA panel was formed from TBEV strains isolated in various regions of the country in different years (Institute of Poliomyelitis Collection, *n* = 14). The TBEV strains used are listed in Additional file 1: Supplementary Table S1.

### Evaluation of the TBEV AmpPS assay

Assay evaluation was implemented using tick samples that were positive or negative for TBEV. In total, 50 RNA samples isolated from Ixodid ticks (*I. ricinus*, *I. persulcatus*, and *D. reticulatus*) collected in northwestern Russia during field visits (2022–2023) were used (Additional file 2: Supplementary Table S2). Testing was performed via the developed TBEV AmpPS assay and the two most commonly used commercial kits for TBEV diagnostics in Russia. The first was the “TBEV, *B. burgdorferi* s.l., *A. phagocytophillum*, *E. chaffeensis*/*E. muris*-FL” kit (AmpliSens, Moscow, Russia). Hereafter, it will be referred to as “commercial kit A.” The second was the “RealBest DNA *Borrelia burgdorferi* s.l./RNA TBEV” kit (AO Vector-Best, Novosibirsk, Russia). Hereafter, it will be referred to as “commercial kit B.”

### Target fragment sequencing

TBEV-positive samples were sequenced on the Illumina MiSeq high-throughput sequencing platform (Illumina, CA, USA) via the MiSeq Reagent Kit v.3 (Illumina, CA, USA). Library preparation was carried out via the KAPA RNA Hyper Prep Kit (Roche, USA) following the manufacturer’s recommendations. The total RNA concentration was measured with a Qubit 4.0 fluorimeter and a Qubit RNA HSAssay Kit (Invitrogen, Waltham, MA, USA) [28].

### Statistical analysis

The 95% confidence interval for a proportion was calculated according to R. Newcombe derived from a procedure outlined by E. Wilson [48, 49] using online GIGA calculator [50].

## Results

Multiple alignment of available TBEV sequences (NCBI GenBank as of December 2024) allowed us to identify a sufficiently conserved region for specific primer and probe design (Table 1). On the basis of these data, oligonucleotides (primers and fluorescent probes) were designed and synthesized, and the TBEV AmpPS assay was developed. The assay includes all the components required for RT‒PCR. An advantage of this assay is that it allows verification of all steps of the analysis, including extraction, reverse transcription, and PCR. In addition, by using EC^−^ and C^−^, the risk of false-positive results due to cross-contamination is minimized (Table 4). In silico analysis of the primer and probe sequences revealed an absence of significant substitutions in the target regions of the TBEV sequences available in GenBank (Fig. 1).

**Table 4:** Overview of the TBEV AmpPS assay

Format	Real-time RT–PCR assay
Components	TBEV super mix, RT–PCR enzyme mix, 2× RT–PCR buffer, armored internal control (IC), armored positive RNA control (ARC^+^), positive PCR control (C^+^), negative control of extraction (CE^−^) and negative control of PCR (C^−^)
Virus detected	*Orthoflavivirus encephalitidis*, tick-borne encephalitis virus (TBEV)
Gene target	*NS3* *gene*
Real-Time PCR platform	CFX96 C1000 Touch (BioRad, USA), Rotor-Gene Q (Qiagen, Germany)
Nucleic acid extraction required	Yes
Suitable specimens for testing	Cell culture, ticks
Sensitivity	10^3^copies/mL (CFX96 C1000 Touch); 10^3^–2.5 × 10^3^ copies/mL (Rotor-Gene Q)
Duration of the analysis (excluding extraction time)	1 h 15 min

**Fig. 2:**
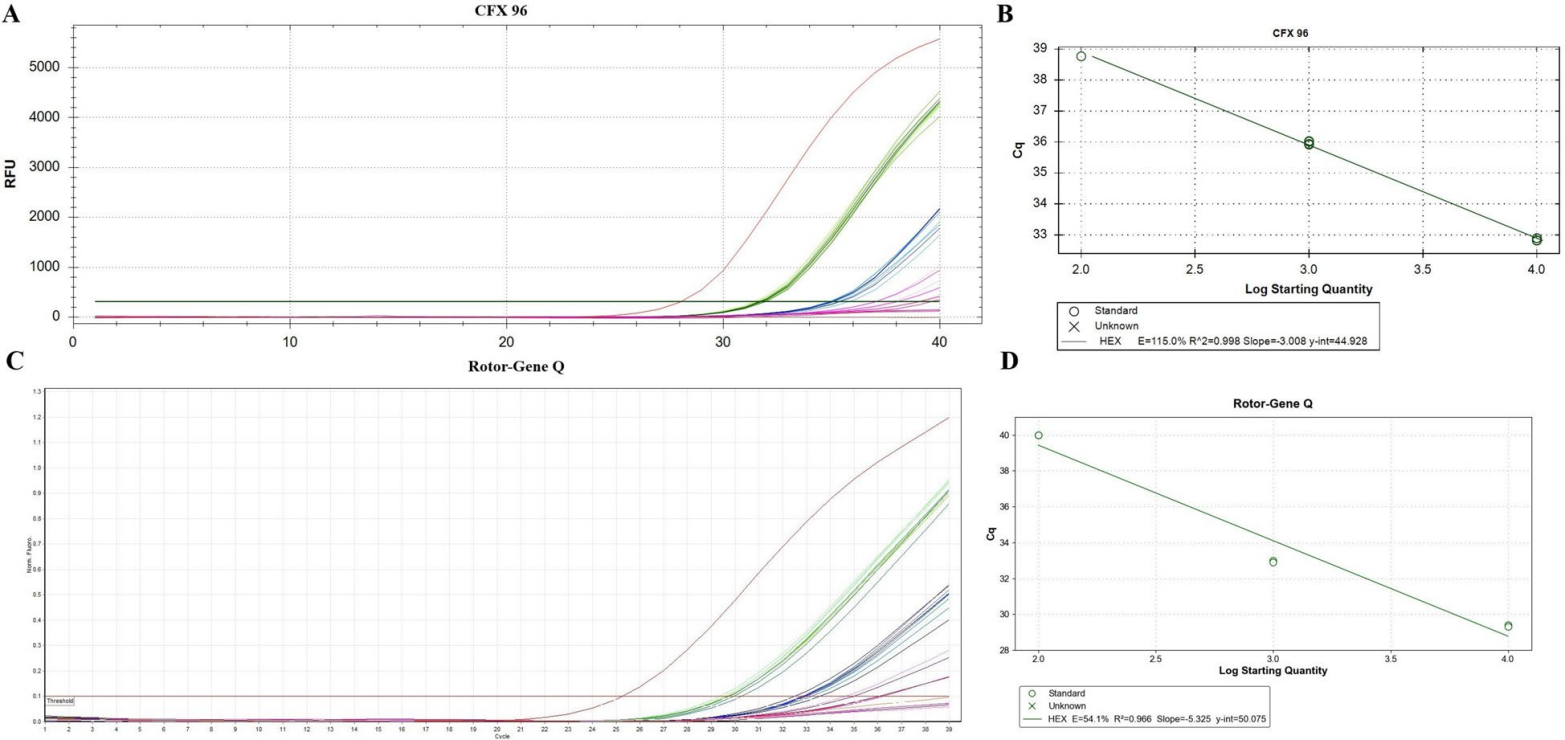
LOD analysis. **A** CFX96 C1000 Touch (Bio-Rad, Hercules, CA, USA) standard curve; **B** LOD assay (CFX96 C1000 Touch (Bio-Rad, USA); **C** Rotor-Gene Q (Rotor-Gene Q, Qiagen, Germany) standard curve; **D** LOD assay Rotor-Gene Q (Rotor-Gene Q, Qiagen, Germany). Samples: red—C^+^, green—ARC 10^4^ copies/ml, blue—ARC 10^3^ copies/mL, pink—ARC 10^2^ copies/mL, and brown—C^−^

The LOD was assessed via ARC dilutions on two PCR machines: a plate (Bio-Rad CFX96 C1000 Touch, USA) and a rotor (Rotor-Gene Q, Qiagen, Germany). The LOD of the assay on the CFX96 and Rotor-Gene Q devices, measured as the maximum dilution yielding a detectable positive signal in all replicates, was 10^3^ RNA copies per mL, with median *C*_t_ values 35.2 (29.5–40.9, 95% confidence intervals (CI)) and 33.2 (27.5–38.9, 95% CI) respectively (Fig. 2, Table 5).

**Table 5:** Threshold cycles (*C*_t_) of the HEX/yellow channel (ARC dilution) at concentrations of 10^4^, 10^3^, 10^2^, copies per ml

PCR machine	Replicates, *C*_t_ values	ARC concentration (copies/mL)				LOD (copies/mL)
		10^4^	10^3^	10^2^	10^1^	
CFX96 C1000 Touch (Bio-Rad, Hercules, CA, USA)	1	31.7	35.4	Negative	Negative	10^3^
	2	31.7	35.0	39.3	Negative	
	3	31.8	35.2	39.7	Negative	
	median	31.7	35.2	Negative	Negative	
	95% CI	26.0–37.4	29.5–40.9	NA	NA	
Rotor-Gene Q (Qiagen, Hilden, Germany)	1	29.9	33.3	39.3	Negative	10^3^
	2	30.0	33.1	Negative	Negative	
	3	30.3	33.2	40.0	Negative	
	Median	30.1	33.2	Negative	Negative	
	95% CI	24.4–35.8	27.5–38.9	NA	NA	

The potential for cross-reactivity was assessed via RNA/DNA from 29 viral and bacterial species belonging to 16 families and 19 genera. No positive reactions were detected with the TBEV AmpPS real-time RT‒PCR assay (Table 3).

The ability of the TBEV AmpPS assay to detect strains prevalent in Russia was shown via a panel of 14 RNAs from TBEV strains isolated in different years in various regions of the country (Additional file 1: Supplementary Table S1). No false-negative results were observed with the developed assay or the commercial kits tested. Consequently, the analytical specificity was 100%. The *C*_t_ values (Additional file 1: Supplementary Table S1) were 12.2–38.7 with median *C*_t_ value 21.3 (18.7–23.9, 95% CI) for the TBEV AmpPS assay, 16.8–42.5 with median *C*_t_ value 19.7 (17.1–22.3, 95% CI) for commercial kit A, and 12.2–29.9 with median *C*_t_ value 26.2 (23.6–28.8, 95% CI) for commercial kit B.

**Fig. 3:**
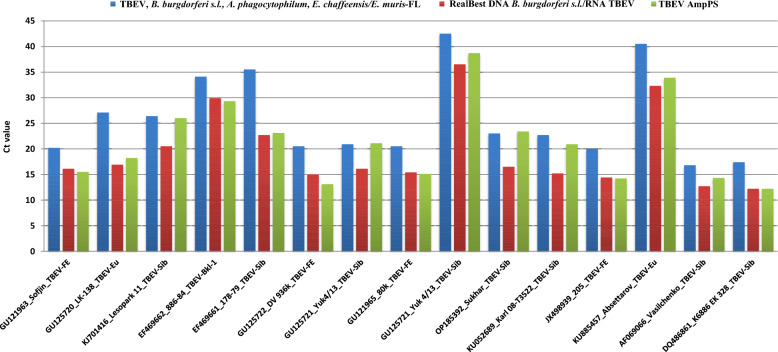
Cycle threshold value (*C*_t_) of the TBEV strain with different test systems. The following systems were used: commercial kit A (“TBEV, *B. burgdorferi* s.l., *A. phagocytophillum*, *E. chaffeensis*/*E. muris*-FL kit,” AmpliSens, Moscow, Russia) (blue); commercial kit B (“RealBest DNA *Borrelia burgdorferi* s.l./RNA TBEV kit,” AO Vector-Best, Russia) (red); and the developed TBEV AmpPS assay (green)

However, our study revealed different sensitivities for different TBEV strains. By comparing the *C*_t_ values of the test systems (Fig. 3), several assumptions can be made. Compared with commercial kit B, TBEV AmpPS appears to be highly sensitive (comparable or slightly better) for the TBEV-FE and TBEV-Sib^*Balt*^ strains and more sensitive in comparison with commercial kit A. TBEV AmpPS appears to be slightly less sensitive than commercial kit B and more sensitive than commercial kit A for the TBEV-Eu and several TBEV-Sib strains (except for TBEV-Sib^*Balt*^). Similar to commercial kit A, TBEV AmpPS appears to be significantly less sensitive than commercial kit B is for several TBEV-Sib strains (except TBEV-Sib^*Balt*^). In silico analysis of partial TBEV nucleotide sequences (*NS3* *genes*) used in the study for target design (available GenBank sequences) revealed the presence of single substitutions in the primer and probe sequences (Additional file 3: Supplementary Fig. S1).

**Fig. 4:**
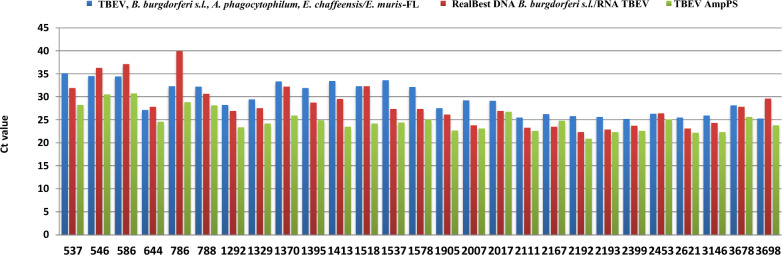
Cycle threshold value (*C*_t_) of RNA from TBEV-positive ticks via different test systems. The following systems were used: commercial kit A (“TBEV, *B. burgdorferi* s.l., *A. phagocytophillum*, *E. chaffeensis*/*E. muris*-FL kit,” AmpliSens, Moscow, Russia) (blue); commercial kit B (“RealBest DNA *Borrelia burgdorferi* s.l./RNA TBEV kit,” AO Vector-Best, Russia) (red); and the developed TBEV AmpPS assay (green)

Simultaneous examination of RNA from tick suspensions via all kits revealed 27 TBEV-positive samples and 23 TBEV-negative concordant samples. The *C*_t_ values (Fig. 4) were as follows: TBEV AmpPS ranged from 30.7–20.9 with median *C*_t_ value 24.8 (23.0–26.8, 95% CI); commercial kit A ranged from 35.1–25.2 with median *C*_t_ value 29.4 (27.6–31.3, 95% CI); and commercial kit B ranged from 39.9–22.3 with median *C*_t_ value 28.1 (26.2–30.0, 95% CI).

In addition, TBEV AmpPS assay-positive samples were partially sequenced successfully in the target region of the assay. In silico analysis of the corresponding primer and probe sequences in the viral genomes revealed an absence of significant substitutions in the target regions of the TBEV isolates from northwestern Russia (Additional file 4: Supplementary Fig. S2).

## Discussion

Among arboviral infections, TBE represents the greatest burden in northern Eurasia and is associated with both increased infection risk and mortality [51]. Globalization and climate change increase the risk of imported TBE cases occurring in nonendemic countries, including the possible establishment of TBEV in previously nonendemic areas. Therefore, rapid, sensitive and specific methods for TBEV detection are needed for the diagnosis, surveillance, and identification of viral sources in the environment following the One Health approach.

Here, we report a new real-time RT‒PCR assay for TBEV detection. We paid special attention to TBEV-Sib^*Balt*^, which is prevalent in northwest Russia. The Saint Petersburg Pasteur Institute is responsible for the TBEV surveillance program in this region. A summary of the developed assay is presented in Table 4. The conditions for the real-time RT‒PCR assay were initially optimized using purified RNA from modified MS2 phage particles. Suitable conditions for PCR with real-time detection were determined for the selected primers and probes. The LOD of the developed assay assessed via both the CFX96 C1000 Touch (Bio-Rad, Hercules, CA, USA) and the Rotor-Gene Q (Qiagen, Hilden, Germany) PCR machines was 10^3^ copies/mL (20 copies/reaction) (Table 5). This approach is valuable for real-time detection of diagnostic kits in the RT‒PCR format [52, 53].

The absence of nonspecific reactions with nucleic acids of other pathogens was determined via a panel of heterologous RNA/DNA from 18 viral and bacterial species, including representatives of 10 viral families (*n* = 29). Comparison of the assay with two commercial kits (commercial kit A and commercial kit B), which used a panel of 14 RNA samples from TBEV strains and RNA from tick suspensions, revealed full convergence of the test results (no discordant samples). Thus, all three systems could be considered suitable for TBEV detection.

However, several differences in sensitivity were observed depending on the TBEV strain. For example, the TBEV AmpPS assay is highly sensitive for the TBEV-FE and TBEV-Sib^*Balt*^ strains, slightly less sensitive for the TBEV-Eu strains and some TBEV-Sib strains (except TBEV-Sib^*Balt*^), and significantly less sensitive for some TBEV-Sib strains (except TBEV-Sib^*Balt*^). In addition, testing of TBEV-positive ticks from northwestern Russia via *C*_t_ shift revealed that the new assay is more sensitive than commercial kit B (average Δ*C*_t_ = 3.3) and more sensitive than commercial kit A (average Δ*C*_t_ = 4.9). Such reduced sensitivity can lead to false-negative results, especially with samples featuring a low TBEV load.

The results obtained generally look logical, taking into account our sequence analysis (assay target region) of TBEV strains and positive samples (northwestern ticks). Although the detected primer and probe mismatches do not critically impact the assay, most of them were indeed found in strains associated with lower sensitivity. Sequence analysis of TBEV from tick samples collected in Northwest Russia revealed that the assay is compatible in design. There were no mismatches in the probe sequence, and at least one primer pair was without substitution in all cases because of the multiplex design.

A comparison of the developed assay and commercial TBEV diagnostic kits used here is presented in Additional file 5: Supplementary Table S3. Unlike our assay, the commercial kits we designed to simultaneously identify two (commercial kit B) or four (commercial kit A) pathogens transmitted by Ixodes ticks. On the one hand, this approach is convenient for studying the diversity of tick-borne infections. On the other hand, such kits are unnecessarily difficult to apply and interpret, especially if the researcher is focused mainly on TBEV. Moreover, the TBEV AmpPS assay is more rapid than commercial kit A is, especially in comparison with commercial kit A, which requires a preliminary reverse transcription step prior to real-time PCR.

Nevertheless, our study shows that the developed assay, similar to the two commercial kits, could be used to detect TBEV genetic variants prevalent in Northwest Russia and likely prevalent in the Baltic countries. Additionally, the developed assay, as well as the two commercial kits, may also be useful for the detection of TBEV isolates prevalent in Siberia and the Russian Far East.

## Conclusions

Here, we report the development and evaluation of the TBEV AmpPS real-time RT‒PCR assay for tick-borne encephalitis virus (*Orthoflavivirus encephalitidis*) detection, which targets a fragment of the *NS3 *gene. The assay contains all of the necessary components to perform analysis. An advantage of this assay is that it allows verification of all analytic steps (extraction, reverse transcription, and PCR). Our results revealed that the LOD of the assay was 10^3^ copies/mL (20 copies/reaction). The developed TBEV AmpPS assay demonstrated high analytical sensitivity, comparable to existing commercial kits, and 100% analytical specificity when tested against a panel tick-borne encephalitis virus strains. The assay can be effectively used for the detection of TBEV-Sib^Balt^ strains both in the course of epidemiological surveillance and for diagnostic purposes. This method is relevant for northwestern Russia, as well as for several European countries where the Siberian subtype circulates, particularly the Baltic states, Finland, the Czech Republic, Slovakia, and the eastern regions of Poland and Germany. As mentioned, TBEV is widespread in Russia, and the burden of TBE among other tick-borne infections is the highest. These conditions necessitate the organization of a continuous surveillance program by public health authorities. The availability of molecular techniques for TBEV diagnostics (mainly real-time PCR-based) is a necessary component, and the availability of such methods enables implementation of the program. Various techniques have been developed, have proven themselves well, and are still being used in routine surveillance. However, several are relatively old. As such, they do not take into account new data on TBEV genetic diversity obtained in recent decades. This could potentially lead to false-negative results in studies. In this context, it is necessary to periodically update the sequences of specific primers and probes on which diagnostic kits are based, taking into account up-to-date data on TBEV genetic diversity. We have endeavored here to design a system that is convenient and applicable while keeping in mind currently relevant strains. Hopefully, the TBEV AmpPS system will play a role in reducing the public health burden associated with *Orthoflavivirus encephalitidis*.

## Supplementary Information

Supplementary Material. 1. Table S1. Positive sample panel used to assess ability of TBEV AmpPS assay to detect different genovariants of TBEV, prevalent in Russia.

Supplementary Material 2. Table S2. Samples used to assess the diagnostic sensitivity of the TBEV AmpPS assay.

Supplementary Material 3. Fig. S1. Partial sequence alignment (*NS3* gene) of the TBEV strains used to assess the ability of the TBEV AmpPS assay to detect different published genetic variants (GenBank), those prevalent in Russia, primers, and probes. Orthoflavivirus encephalitis sequences with the following GenBank accession numbers were aligned: JX498939 (TBEV-FE), EF469661 (TBEV-Sib), EF469662 (TBEV-Bkl1), KJ701416 (TBEV-Sib), AF069066 (TBEV-Sib), DQ486861 (TBEV-Sib) and KU885457 (TBEV-Eu).

Supplementary Material 4. Fig. S2. Partial sequence alignment (*NS3 *gene), including TBEV isolate sequences used in the study, primers, and probes. Orthoflavivirus encephalitidis isolate sequences with the following sample IDs were aligned: 537, 546, 586, 644, 786, 788, 1292, 1329, 1370, 1395, 1413, 1518, 1537, 1578, 1905, 2007, 2017, 2111, 2167, 2192, 2193, 2399, 2453, 2621, 3146, 3678 and 3698 (Additional file 5: Table S3).

Supplementary Material 5. Table S3. Comparison of kits/assay for TBEV diagnostics, used in the study.

## Data Availability

Data supporting the main conclusions of this study are included in the manuscript.
