# Supplementary material for: Development and evaluation of an assay for the detection of tick-borne encephalitis virus RNA via real-time PCR with reverse transcription
Source: Parasit Vectors. 2026 Mar 23;19:191. doi: 10.1186/s13071-026-07366-5 (PMC13130820; doi:10.1186/s13071-026-07366-5)
Supplement: Supplementary file 1 — Supplementary Material. 1. Table S1. Positive sample panel used to assess ability of TBEV AmpPS assay to detect different genovariants of TBEV, prevalent in Russia. [file 13071_2026_7366_MOESM1_ESM.docx]

Additional file 1: Table S1. Positive sample panel used to assess ability of TBEV AmpPS assay to detect different genovariants of TBEV, prevalent in Russia

| N | Strain | Lg PFU/mL | GenBank NCBI ID | Genotype | Isolation data | Host | RealBest DNA Borrelia burgdorferi s.l./RNA TBEV (©AO Vector-Best, Novosibirsk, Russia),  Ct value | TBEV, B. burgdorferi s.l., *A. phagocytophillum*, *E. chaffeensis*/*E. muris*-FL (AmpliSens^®^, Moscow, Russia),  Ct value | TBEV AmpPS (Pasteur Institute, SPb, Russia),  Ct value |
| --- | --- | --- | --- | --- | --- | --- | --- | --- | --- |
| 1 | Sofjin | 6.0 | GU121963 | TBEV-FE | 1937 | Brain of the patient with acute TBE; cloned | 16.1 | 20.2 | 15.5 |
| 2 | LK-138 | 6.0 | GU125720 | TBEV-Eu | 1972 | 30 male imago ticks *I. ricinus* | 16.9 | 27.1 | 18.2 |
| 3 | Lesopark 11 | 7.0 | KJ701416 | TBEV-Sib | 1986 | Imago ticks *I. persulcatus* | 20.5 | 26.4 | 26.0 |
| 4 | 886-84 | 6.7 | EF469662 | TBEV-Bkl-1 | 1984 | Clethrionomys rufocanus,  brain tissue lysate | 29.9 | 34.1 | 29.3 |
| 5 | 178-79 | 8.0 | EF469661 | TBEV-Sib | 1979 | *I. persulcatus* | 22.7 | 35.5 | 23.1 |
| 6 | DV 936k | 6.0 | GU125722 | TBEV-FE | 1975 | Imago ticks *H. concina* | 15.0 | 20.5 | 13.1 |
| 7 | 80k | NA | GU121965 | TBEV-FE | before 1970 | Blood of the patient with TBE; cloned | 15.4 | 20.5 | 15.1 |
| 8 | Yuk 4/13 | NA | GU125721 | TBEV-Sib | 1969 | Imago tick *I. persulcatus* | 36.5 | 42.5 | 38,7 |
| 9 | Sukhar | 7.0 | OP185392 | TBEV-Sib | 2012 | Brain of deceased  TBE patient | 16.5 | 23.0 | 23.4 |
| 10 | Karl 08-T3522 | 7.5 | KU052689 | TBEV-Sib | 2008 | Pool of *I. Persulcatus* | 15.2 | 22.7 | 20.9 |
| 11 | 205 | 5.5 | JX498939 | TBEV-FE | 1973 | Imago ticks *I. persulcatus* | 14.4 | 20.0 | 14.2 |
| 12 | Absettarov | 6.8 | KU885457 | TBEV-Eu | 1951 | Blood of the patient with TBE | 32.3 | 40.5 | 33.9 |
| 13 | Vasilchenko | 7.6 | AF069066 | TBEV-Sib | 1961 | Blood of a patient with acute TBE | 12.7 | 16.8 | 14.3 |
| 14 | EK 328 | NA | DQ486861 | TBEV-Sib | 1972 | Imago ticks *I. persulcatus* | 12.2 | 17.4 | 12.2 |
| Median C_t_ value | | | | | | | 19.7 | 26.2 | 21.3 |
| 95% CI | | | | | | | 17.1-22.3 | 23.6-28.8 | 18.7-23.9 |
