# Supplementary material for: Development and evaluation of an assay for the detection of tick-borne encephalitis virus RNA via real-time PCR with reverse transcription
Source: Parasit Vectors. 2026 Mar 23;19:191. doi: 10.1186/s13071-026-07366-5 (PMC13130820; doi:10.1186/s13071-026-07366-5)
Supplement: Supplementary file 2 — Supplementary Material 2. Table S2. Samples used to assess the diagnostic sensitivity of the TBEV AmpPS assay. [file 13071_2026_7366_MOESM2_ESM.docx]

Additional file 2: Table S2. Samples used to assess the diagnostic sensitivity of the TBEV AmpPS assay

| N | Sample ID | Tick species | Region of sampling | Collection date | TBEV, *B.burgdorferi* s.l., *A.phagocytophilum, E.chaffeensis* / *E.muris*-FL (AmpliSens^®^, Moscow, Russia), (commercial kit A)  Ct value | RealBest DNA *Borrelia burgdorferi* s.l./RNA TBEV (©AO Vector-Best, Novosibirsk, Russia), (commercial kit B)  Ct value | TBEV AmpPS (Pasteur Institute, SPB, Russia),  Ct value |
| --- | --- | --- | --- | --- | --- | --- | --- |
| 1 | 537 | *I.persulcatus* | Arkhangelsk Region | 2022 | 35.1 | 31.9 | 28.2 |
| 2 | 546 | *I.persulcatus* | Arkhangelsk Region | 2022 | 34.5 | 36.3 | 30.5 |
| 3 | 586 | *I.persulcatus* | Arkhangelsk Region | 2022 | 34.4 | 37.1 | 30.7 |
| 4 | 644 | *I.persulcatus* | Republic of Karelia | 2022 | 27.1 | 27.8 | 24.6 |
| 5 | 786 | *I.persulcatus* | Republic of Karelia | 2022 | 32.3 | 39.9 | 28.8 |
| 6 | 788 | *I.persulcatus* | Leningrad Region | 2022 | 32.2 | 30.6 | 28.1 |
| 7 | 1292 | *I.persulcatus* | Leningrad Region | 2022 | 28.2 | 26.9 | 23.4 |
| 8 | 1329 | *I.persulcatus* | Leningrad Region | 2022 | 29.4 | 27.5 | 24.2 |
| 9 | 1370 | *I.persulcatus* | Leningrad Region | 2022 | 33.3 | 32.2 | 25.9 |
| 10 | 1395 | *I.persulcatus* | Leningrad Region | 2022 | 31.9 | 28.7 | 24.9 |
| 11 | 1413 | *I.persulcatus* | Leningrad Region | 2022 | 33.4 | 29.5 | 23.5 |
| 12 | 1518 | *I.persulcatus* | Leningrad Region | 2022 | 32.3 | 32.3 | 24.2 |
| 13 | 1537 | *I.persulcatus* | Leningrad Region | 2022 | 33.6 | 27.3 | 24.4 |
| 14 | 1578 | *I.persulcatus* | Leningrad Region | 2022 | 32.1 | 27.3 | 25.0 |
| 15 | 1905 | *I.persulcatus* | Leningrad Region | 2022 | 27,5 | 26.1 | 22.7 |
| 16 | 2007 | *I.persulcatus* | Leningrad Region | 2023 | 29.2 | 23.8 | 23.1 |
| 17 | 2017 | *I.persulcatus* | Leningrad Region | 2023 | 29.1 | 26.9 | 26.7 |
| 18 | 2111 | *I.persulcatus* | Leningrad Region | 2023 | 25.5 | 23.3 | 22.6 |
| 19 | 2167 | *I.persulcatus* | Leningrad Region | 2023 | 26.2 | 23.5 | 24.8 |
| 20 | 2192 | *I.persulcatus* | Leningrad Region | 2023 | 25.8 | 22.3 | 20.9 |
| 21 | 2193 | *I.persulcatus* | Leningrad Region | 2023 | 25.6 | 22.9 | 22.3 |
| 22 | 2399 | *I.persulcatus* | Leningrad Region | 2023 | 25.2 | 23.7 | 22.6 |
| 23 | 2453 | *I.persulcatus* | Leningrad Region | 2023 | 26.3 | 26.4 | 25.1 |
| 24 | 2621 | *I.persulcatus* | Leningrad Region | 2023 | 25.5 | 23.1 | 22.2 |
| 25 | 3146 | *D.reticulatus* | Leningrad Region | 2023 | 25.9 | 24.3 | 22.3 |
| 26 | 3678 | *I.ricinus* | Leningrad Region | 2023 | 28.1 | 27.8 | 25.6 |
| 27 | 3698 | *I.ricinus* | Leningrad Region | 2023 | 25.3 | 29.6 | 23.8 |
| Median C_t_ value | | | | | 29.4 | 28.1 | 24.8 |
| 95% CI | | | | | 27.6-31.3 | 26.2-30.0 | 23.0-26.8 |
| 1 | 571 | *I.persulcatus* | Arkhangelsk Region | 2022 | negative | negative | negative |
| 2 | 572 | *I.persulcatus* | Arkhangelsk Region | 2022 | negative | negative | negative |
| 3 | 573 | *I.persulcatus* | Arkhangelsk Region | 2022 | negative | negative | negative |
| 4 | 602 | *I.persulcatus* | Arkhangelsk Region | 2022 | negative | negative | negative |
| 5 | 603 | *I.persulcatus* | Arkhangelsk Region | 2022 | negative | negative | negative |
| 6 | 604 | *I.persulcatus* | Arkhangelsk Region | 2022 | negative | negative | negative |
| 7 | 605 | *I.persulcatus* | Arkhangelsk Region | 2022 | negative | negative | negative |
| 8 | 1256 | *I.ricinus* | Leningrad Region | 2022 | negative | negative | negative |
| 9 | 1257 | *I.ricinus* | Leningrad Region | 2022 | negative | negative | negative |
| 10 | 1265 | *I.persulcatus* | Leningrad Region | 2022 | negative | negative | negative |
| 11 | 2943 | *I.ricinus* | Leningrad Region | 2023 | negative | negative | negative |
| 12 | 2956 | *I.persulcatus* | Leningrad Region | 2023 | negative | negative | negative |
| 13 | 2773 | *I.persulcatus* | Leningrad Region | 2023 | negative | negative | negative |
| 14 | 2774 | *I.ricinus* | Leningrad Region | 2023 | negative | negative | negative |
| 15 | 2918 | *I.ricinus* | Leningrad Region | 2023 | negative | negative | negative |
| 16 | 661 | *I.persulcatus* | Republic of Karelia | 2022 | negative | negative | negative |
| 17 | 662 | *I.persulcatus* | Republic of Karelia | 2022 | negative | negative | negative |
| 18 | 1428 | *I.ricinus* | Republic of Karelia | 2022 | negative | negative | negative |
| 19 | 3728 | *I.persulcatus* | Republic of Karelia | 2023 | negative | negative | negative |
| 20 | 3729 | *I.persulcatus* | Republic of Karelia | 2023 | negative | negative | negative |
| 21 | 3737 | *I.persulcatus* | Republic of Karelia | 2023 | negative | negative | negative |
| 22 | 3753 | *I.persulcatus* | Republic of Karelia | 2023 | negative | negative | negative |
| 23 | 3754 | *I.persulcatus* | Republic of Karelia | 2023 | negative | negative | negative |
