# Supplementary figures and images for: Development and evaluation of an assay for the detection of tick-borne encephalitis virus RNA via real-time PCR with reverse transcription
Source: Parasit Vectors. 2026 Mar 23;19:191. doi: 10.1186/s13071-026-07366-5 (PMC13130820; doi:10.1186/s13071-026-07366-5)

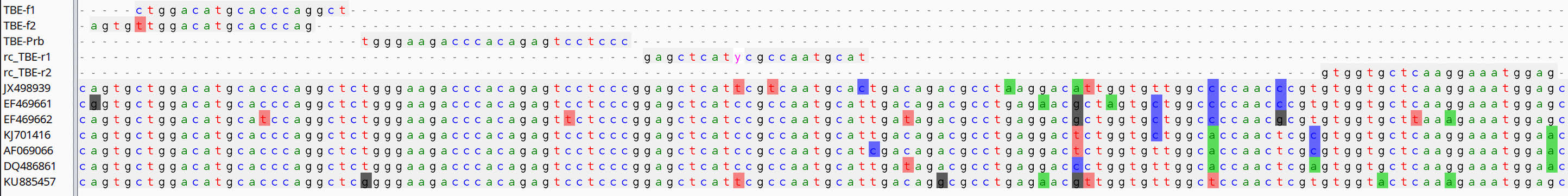

Supplement: Supplementary file 3 — Supplementary Material 3. Fig. S1. Partial sequence alignment (NS3 gene) of the TBEV strains used to assess the ability of the TBEV AmpPS assay to detect different published genetic variants (GenBank), those prevalent in Russia, primers, and probes. Orthoflavivirus encephalitis sequences with the following GenBank accession numbers were aligned: JX498939 (TBEV-FE), EF469661 (TBEV-Sib), EF469662 (TBEV-Bkl1), KJ701416 (TBEV-Sib), AF069066 (TBEV-Sib), DQ486861 (TBEV-Sib) and KU885457 (TBEV-Eu). [file 13071_2026_7366_MOESM3_ESM.jpg]

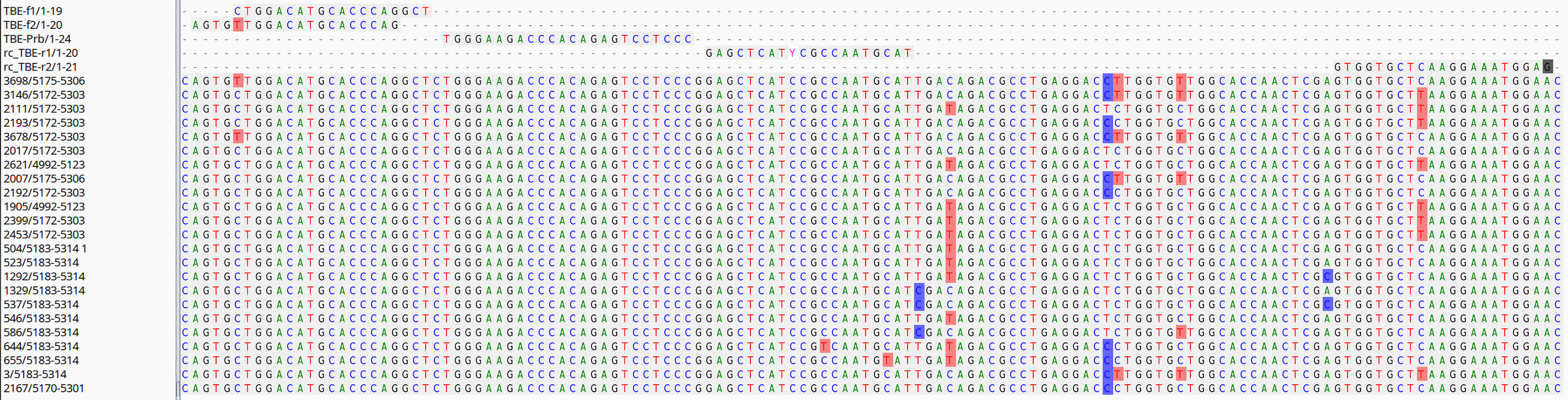

Supplement: Supplementary file 4 — Supplementary Material 4. Fig. S2. Partial sequence alignment (NS3 gene), including TBEV isolate sequences used in the study, primers, and probes. Orthoflavivirus encephalitidis isolate sequences with the following sample IDs were aligned: 537, 546, 586, 644, 786, 788, 1292, 1329, 1370, 1395, 1413, 1518, 1537, 1578, 1905, 2007, 2017, 2111, 2167, 2192, 2193, 2399, 2453, 2621, 3146, 3678 and 3698 (Additional file 5: Table S3). [file 13071_2026_7366_MOESM4_ESM.jpg]
