# Supplementary material for: Development and evaluation of an assay for the detection of tick-borne encephalitis virus RNA via real-time PCR with reverse transcription
Source: Parasit Vectors. 2026 Mar 23;19:191. doi: 10.1186/s13071-026-07366-5 (PMC13130820; doi:10.1186/s13071-026-07366-5)
Supplement: Supplementary file 5 — Supplementary Material 5. Table S3. Comparison of kits/assay for TBEV diagnostics, used in the study. [file 13071_2026_7366_MOESM5_ESM.docx]

Additional file 5: Table S3. Comparison of kits/assay for TBEV diagnostics, used in the study

| Kit/assay name | RealBest DNA *B. burgdorferi* s.l./RNA TBEV | TBEV, *B.burgdorferi* s.l., *A.phagocytophilum*, *E.chaffeensis* / *E.muris*-FL | TBEV AmpPS assay |
| --- | --- | --- | --- |
| Manufacturer | ©AO Vector-Best, Novosibirsk, Russia | AmpliSens, Moscow, Russia | Saint Petersburg Pasteur Institute, Russia |
| Kit/assay format | One step real-time RT-PCR Kit | Two step real-time PCR Kit | One step real-time RT-PCR assay |
| Kit size | 48 | 60 | 100 |
| Pathogens detected | *B. burgdorferi* s.l., TBEV | TBEV, *B.burgdorferi* s.l., *A.phagocytophilum*, *E.chaffeensis* / *E.muris* | TBEV |
| Gene Target | NA | 16S (*B.burgdorferi* s.l.), msp2 (*A.phagocytophilum*), 16S (*E.chaffeensis*/*E.muris*), C (TBEV) | NS3 |
| LOD | 100 copies per reaction | 5х10^3^ copies/mL | 10^3^ copies/mL |
| Reaction volume | 50 | 25 | 25 |
| Kit/assay components |  | PCR-mix-1-FRT TBEV, A. ph., E. ch./E. m.; PCR-mix-1-FRT B. b.s.l./IC, PCR-buffer-C, TaqF polymerase, positive PCR control (C+) cDNA TBEV, B. b.s.l., A. ph., E.ch./E.m./IC, armored internal control (IC), negative control of extraction (CE-) and negative control of PCR (C-) | TBEV super mix, RT-PCR enzyme mix, 2-x RT-PCR buffer, armored internal control (IC), armored positive RNA control (ARC+), positive PCR control (C+), negative control of extraction (CE-) and negative control of PCR (C-) |
| Laboratory instruments required but not provided with Kit/assay | Real time PCR platform Specify instruments: iQ iCycler (Bio-Rad, USA), iQ5 iCycler (Bio-Rad, USA), CFX96 C1000 Touch (Bio-Rad, USA), DT-96 (DNA-Technology, Russia).  Desktop centrifuge, Plate centrifuge, Vortex mixer, Micropipettes, Barrier pipette tips, Powder free gloves, 96 well reaction plates, Nuclease-Free Water, Biosafety Level 2 laboratory | Real time PCR platform Specify instruments: - Rotor-Gene 6000 Corbett Research, Australia Rotor-Gene Q (Qiagen, Germany) CFX96 (Bio-Rad, USA) DT-praim (DNA-Technology, Russia).  Desktop centrifuge, Plate centrifuge, Vortex mixer, Micropipettes, Barrier pipette tips, Powder free gloves, 96 well reaction plates, Nuclease-Free Water, Biosafety Level 2 laboratory | Real time PCR platform Specify instruments: -  CFX96 C1000 Touch (Bio-Rad, USA), Rotor-Gene Q (Qiagen, Germany). Desktop centrifuge, Plate centrifuge, Vortex mixer, Micropipettes, Barrier pipette tips, Powder free gloves, 96 well reaction plates, Nuclease-Free Water, Biosafety Level 2 laboratory |
| Other materials required but not provided | RealBest extraction 100 Kit (©AO Vector-Best, Novosibirsk, Russia) | RIBO-prep kit (AmpliSens^®^, Moscow, Russia), Magno-sorb (AmpliSens^®^, Moscow, Russia) | RIBO-prep kit (AmpliSens^®^, Moscow, Russia) |
| Laboratory space | Unidirectional workflow; laboratory area must contain dedicated workspace and equipment for specimen extraction, preparation of mastermix and amplification | Unidirectional workflow; laboratory area must contain dedicated workspace and equipment for specimen extraction, preparation of mastermix and amplification | Unidirectional workflow; laboratory area must contain dedicated workspace and equipment for specimen extraction, preparation of mastermix and amplification |
| Equipment maintenance | Recommended maintenance and calibration for real time PCR platforms, micropipettes and standard laboratory equipment Recommended maintenance and calibration for real time PCR platforms, micropipettes and standard laboratory equipment Recommended maintenance and calibration for real time PCR platforms, micropipettes and standard laboratory equipment | Recommended maintenance and calibration for real time PCR platforms, micropipettes and standard laboratory equipment Recommended maintenance and calibration for real time PCR platforms, micropipettes and standard laboratory equipment Recommended maintenance and calibration for real time PCR platforms, micropipettes and standard laboratory equipment | Recommended maintenance and calibration for real time PCR platforms, micropipettes and standard laboratory equipment Recommended maintenance and calibration for real time PCR platforms, micropipettes and standard laboratory equipment Recommended maintenance and calibration for real time PCR platforms, micropipettes and standard laboratory equipment |
| Specimen inactivation using enhanced biosafety required by user prior to performing the test | Specimen inactivated by addition of lysis buffer. | Specimen inactivated by addition of lysis buffer. | Specimen inactivated by addition of lysis buffer. |
| Nucleic acid extraction required by user | Yes | Yes | Yes |
| Reverse transcription required by user | No | Yes | No |
| Throughput: Time to results | 1h 36min | 2h 28min | 1h 15min |
| Storage conditions for reagents | 2^°^C - 8^°^C | 2^°^C - 8^°^C (for IC, C-), -16^°^C - -24^°^C (for PCR-mix-1-FRT TBEV, A. ph., E. ch./E. m.; PCR-mix-1-FRT B. b. s.l./IC, PCR-buffer-C, TaqF polymerase, C+) | 2^°^C - 8^°^C (for IC, ARC+, CE-), -16^°^C - -24^°^C (for TBEV super mix, RT-PCR enzyme mix, 2-x RT-PCR buffer, C+, C-) |
